# Decreasing Nitrogen Fertilizer Input Had Little Effect on Microbial Communities in Three Types of Soils

**DOI:** 10.1371/journal.pone.0151622

**Published:** 2016-03-18

**Authors:** Hailing Yu, Qiang Gao, Zeqiang Shao, Anning Ying, Yuyang Sun, Jingwei Liu, Wei Mao, Bin Zhang

**Affiliations:** 1 College of Resources and Environment, Jilin Agricultural University, Changchun, Jilin, China; 2 College of Land and Environment, Shenyang Agricultural University, Shenyang, Liaoning, China; Chinese Academy of Sciences, CHINA

## Abstract

In this study, we examined the influence of different nitrogen (N) application rates (0, 168, 240, 270 and 312 kg N ha^-1^) on soil properties, maize (*Zea mays* L.) yields and microbial communities of three types of soils (clay, alluvial and sandy soils). Phospholipid fatty acid analysis was used to characterize soil microbial communities. Results indicated that N fertilization significantly decreased microbial biomass in both clay and sandy soils regardless of application rate. These decreases were more likely a result of soil pH decreases induced by N fertilization, especially in the sandy soils. This is supported by structural equation modeling and redundancy analysis results. Nitrogen fertilization also led to significant changes in soil microbial community composition. However, the change differences were gradually dismissed with increase in N application rate. We also observed that N fertilization increased maize yields to the same level regardless of application rate. This suggests that farmers could apply N fertilizers at a lower rate (i.e. 168 kg N ha^-1^), which could achieve high maize yield on one hand while maintain soil microbial functions on the other hand.

## Introduction

Application of nitrogen (N) fertilizers into arable fields has been recognized as a critical agricultural practice to increase crop yields [1]. However, excessive use of N fertilizers has resulted in serious soil degradation such as significant acidification of agricultural soils, nitrate leaching and soil organic matter reduction [2,3]. Therefore, it is urgently required to find appropriate application rates of N fertilizers that increase crop productivity while benefit soil quality.

Soil microorganisms have been widely used as indicators of soil biological quality [4,5]. They play critical roles in many soil processes such as organic matter decomposition, nutrient cycling and formation of soil structure [6,7]. There are many researchers who have examined the responses of biomass and composition of soil microbial communities to N fertilization rate [8,9]. Some found that soil microbial biomass was significantly decreased after application of N fertilizers at higher rates [10,11], while others did not report any significant changes. For example, Roberts et al. [12] found no differences in soil microbial biomass between treatments with urea applied at rates of 20 and 130 kg N ha^-1^. Similarly, Lupwayi et al. [13] found no consistent changes in soil microbial biomass after application of urea at rates up to 90 kg N ha^-1^ to no-till barley. The response of soil microbial community structure to N fertilizers varied considerable among studies and no clear trends emerged [14]. It was reported that N fertilization increased the relative abundance of fungi in two European soils [15] but decreased that in a Swedish soil [16]. A few studies further suggested that application of N fertilizers exerted no or only small effects on the composition of soil microbial communities [17]. However, most of these studies were conducted in fields which have quite different climate conditions and soil types. Uncertainties still remain about the response of soil microbial communities to application of N fertilizers in fields which have different soil types but same climate conditions. This kind of information might be helpful in identifying the specific response trend of soil microbes to N fertilization.

In the current study, soils were taken from three fertilization experiments which had same climate and N fertilization treatment but different soil types. The effects of applying different rates of N fertilizers on biomass and composition of microbial communities were examined in three types of soils. We used phospholipid fatty acid analysis (PLFA) to examine soil microbial biomass and community composition [18]. The hypotheses of this study were: 1) N fertilization would lead to significant changes in soil microbial communities and 2) the extent to which of change depends on the application rate of N fertilizers.

## Materials and Methods

### Site description and experimental setup

This study was conducted on three fertilization trials initiated in the spring of 2009 at Sankeshu (43°20′ N, 124°00′ E), Wangjiaqiao (43°15′ N, 124°29′ E) and Fujiajie (43°21′ N, 124°05′ E) in Jilin Province of northeastern China. These three sites are privately owned to local farmers who have signed long-term land contracts with Jilin Agricultural University. The owners of these lands have given permission to conduct the study on their sites. No endangered or protected species were sampled on these sites. The distances among these three sites are less than four kilometers. These sites experience a similar humid continental climate with mean annual air temperature of 5.8°C and mean annual precipitation of 474 mm. The soils of Sankeshu, Wangjiaqiao and Fujiajie are clay, alluvial and sandy soils, respectively, and classified as Chernozems, Fluvisols and Arenosols according to the WRB soil classification system [19]. The soil texture in the top 0.2 m was 325 g sand kg^-1^, 252 g silt kg^-1^ and 423 g clay kg^-1^ for the clay soils, 477 g sand kg^-1^, 296 g silt kg^-1^ and 227 g clay kg^-1^ for the alluvial soils, and 736 g sand kg^-1^, 96 g silt kg^-1^ and 108 g clay kg^-1^ for the sandy soils.

The experimental design was exactly the same for these three sites. The experiment was a randomized complete block design with five treatments and three replicates, which resulted in a total of 15 plots for every site. Each plot was 10 m long and 6 m wide. The five treatments were N application as urea at rates of 0, 168, 240, 270 and 312 kg N ha^-1^. For each treatment, one third of the N fertilizer was applied at planting and the other two third was side-dressed at the maize (*Zea mays* L.) six-leaf stage. Calcium superphosphate and potassium sulfate were applied as basal fertilizers at rates of 100 kg P_2_O_5_ ha^-1^ and 120 kg K_2_O ha^-1^, respectively. All plots were cropped to maize every year. Maize was planted in May at a target population of 65,000 plants ha^-1^ and harvested in October. All maize residues were removed from the plots after harvest. Tillage was performed on all plots to a depth of 0.2 m in the spring of each year with a combination of chisel plow and field cultivator to produce an adequate seed bed. Weeds were controlled by applying herbicides before seedling emergence.

### Soil sampling and analysis

Soil samples were taken with a T sampler from the top 0.2 m depth on 7 Oct 2014 after harvest. For every plot, eight randomized soil cores were sampled and mixed to represent one field replicate. A total of 45 soil samples (five treatments × three replicates × three sites) were collected. These soil samples were placed in plastic bags and transported into laboratory in a cooler. Once in the laboratory, soil samples were passed through a 2-mm sieve and homogenized. All plant materials were removed manually before sieving. The soil samples used for chemical analyses were air-dried. The soil samples used for PLFA analysis were freeze-dried and kept in a desiccator before extraction.

Soil pH was determined by a pH meter with in a soil/water ratio of 1:2.5. Soil organic C contents were determined by the Walk-Black method [20]. Soil total N contents were determined by the micro-Kjeldhal method [21]. Available N was determined by the alkaline hydrolysis diffusion procedure, available phosphorus (P) was determined by the Olsen-P procedure, and available potassium (K) was determined by the ammonium acetate extraction procedure [22]. Maize yields were determined by manually harvesting from an area of 18 m^2^ (six rows with a width of 3.6 m and a length of 5 m) in the middle of each plot at 15.5% moisture content.

### Phospholipid fatty acid analysis

The PLFA extraction method as described by Zhang et al. [23], modified from Bligh and Dyer [24], was followed in this study. In brief, a mixture of chloroform-methanol-citrate (1:2:0.8) was used to extract lipids from soils. Phospholipids were separated from the solution with solid phase extraction columns (CNW Technologies GmbH, Düsseldorf, Germany) and analyzed by a 6890N gas chromatography (Agilent Technologies, Santa Clara, USA). An HP-5 column (30 m × 0.32 mm × 0.25 μm) and flame ionization detector were used. The fatty acids were identified by the MIDI Sherlock Microbial Identification System (Microbial ID Inc., Newark, USA). We used nonadecanoic acid methyl ester as the internal standard.

Total PLFAs were calculated as the sum of all PLFAs detected. We used 18:2ω6,9c to represent fungi [18], the sum of i14:0, a15:0, i15:0, i16:0, a17:0 and i17:0 to represent Gm+ bacteria, the sum of 16:1 2OH, 16:1ω7c, 16:1ω9c, cy17:0, 17:1ω8c, 18:1ω7c and cy19:0 to represent Gram-negative (Gm-) bacteria [25] and the sum of 10Me16:0, 10Me17:0 and 10Me18:0 to represent actinomycetes.

### Statistical analysis

General linear model analysis of variance was conducted to test the effect of N fertilization rate and soil type on soil properties, maize yields and sums and ratios of specific microbial groups. Normality assumption and homogeneity of variance were checked with Shapiro-Wilk and Bartlett’s tests, respectively. The Tukey’s honestly significant difference test was used as a post-hoc. Statistical analysis was carried out by SPSS 13.0 for Windows (SPSS Inc., Chicago, USA). Figures were graphed with Sigmaplot 10.0 (Systat Software Inc., San Jose, USA).

Principal component analysis (PCA) was carried out with the PLFA data (mol%) to examine the variation in soil microbial community composition. A non-parametric multivariate analysis of variance was conducted on the Bray-Curtis dissimilarity matrix to examine significant composition differences among treatments with the ‘vegdist’ and ‘adonis’ functions in the ‘vegan’ library of the R program (version 3.2.1). Redundancy analysis (RDA) was also carried out with the ‘rda’ function in the ‘vegan’ library of the R program to examine relationships between soil chemical properties and soil microbial communities. Adjusted R-squared values were used to calculate the proportion of variance explained [26]. Permutation tests were conducted with the ‘anova.cca’ function in R to examine the significance of explanatory variables. The RDA graph was generated by the R program.

Structural equation models were fitted with the ‘sem’ package in R to examine the direct and indirect effect of treatments on soil microorganisms. Explanatory variables included soil pH, soil organic C, and ‘soil fertility’ indicated by total N, the ratio of C to N (C/N), available N, P and K. Bacterial biomass was treated as a latent variable and was indicated by Gm+ and Gm–. We used the following criteria to check significance of model fit: *χ*^*2*^/df < 2, *P* > 0.05, root mean square error of approximation (*RMSEA*) < 0.07, goodness of fit index (GFI) > 0.9 [27].

## Results

### Soil characteristics and maize yields

Application of N fertilizers generally decreased pH values of the three studied soils, especially for the sandy soils which had pH decreases from 0.56 to 0.92 (*P* < 0.05, Table 1). The pH values were higher in the clay soils than the alluvial and sandy soils (*P* < 0.05). Compared with the no fertilization treatment, application of N fertilizers at rate of 168 kg N ha^-1^ significantly decreased organic C content for the alluvial soils, application of N fertilizers at rate of 270 kg N ha^-1^ significantly increased that for the sandy soils, application of N fertilizers at rate of 312 kg N ha^-1^ significantly decreased organic C content for both clay and alluvial soils but increased that for the sandy soils (*P* < 0.05, Table 1). The clay soils contained greater organic C than the alluvial soils, which also contained greater organic C than the sandy soils (*P* < 0.05). Total N was higher (*P* < 0.05) in the clay than the alluvial and sandy soils but not influenced (*P* > 0.05) by the N application rate. For the clay soils, application of N fertilizers at rate of 312 kg N ha^-1^ resulted in significantly lower C/N ratios compared with the other treatments (*P* < 0.05, Table 1). The C/N ratios in the sandy soils were gradually increased with increase in N application rate (Table 1). The clay soils had higher C/N ratios than the alluvial soils, which also had higher C/N ratios than the sandy soils (*P* < 0.05). Compared with the no fertilization treatment, application of N fertilizers at rates of 240, 270 and 312 kg N ha^-1^ led to significantly higher available N contents in the alluvial soils (*P* < 0.05, Table 1). Nitrogen fertilization had no effect on available P and K contents regardless of soil type (*P* > 0.05, Table 1). Available N, P and K contents were highest in the clay soils, intermediate in the alluvial soils, and lowest in the sandy soils (*P* < 0.05). Nitrogen fertilization significantly increased maize yields regardless of soil type (*P* < 0.05, Table 1). However, maize yields were not significantly different among treatments of N applied at rates of 168, 240, 270 and 312 kg N ha^-1^ (*P* > 0.05, Table 1). Maize yields for the clay and alluvial soils were higher than these for the sandy soils (*P* < 0.05).

**Table 1:** Soil characteristics and maize yields (averaged from 2009 to 2012) under application of N fertilizers at five rates.

	N application rate	pH	SOC	TN	C/N	Available N	Available P	Available K	Maize yield
	(kg N ha^-1^)		(g kg^-1^)	(g kg^-1^)		(mg kg^-1^)	(mg kg^-1^)	(mg kg^-1^)	(kg ha^-1^ yr^-1^)
**Clay soil**	0	6.13 (0.03) a†	17.5 (1.82) a	1.43 (0.03) a	12.2 (1.18) a	127 (23.7) a	32.4 (10.7) a	195 (19.3) a	6215 (679) b
	168	5.98 (0.07) ab	16.8 (0.28) a	1.45 (0.06) a	11.6 (0.60) a	129 (10.2) a	38.0 (12.5) a	185 (11.0) a	9882 (368) a
	240	5.96 (0.08) ab	16.7 (0.33) a	1.43 (0.04) a	11.7 (0.47) a	128 (1.85) a	36.2 (8.95) a	203 (20.0) a	10164 (290) a
	270	5.90 (0.07) b	16.3 (0.65) a	1.40 (0.03) a	11.7 (0.64) a	130 (5.35) a	45.6 (24.4) a	175 (17.3) a	10059 (326) a
	312	5.89 (0.10) b	13.6 (0.45) b	1.43 (0.05) a	9.54 (0.60) b	136 (1.40) a	32.3 (7.27) a	185 (16.2) a	9773 (463) a
**Alluvial soil**	0	5.80 (0.08) a	12.9 (0.90) a	1.18 (0.03) a	10.9 (1.01) a	86.6 (2.83) b	24.5 (11.5) a	173 (24.9) a	7873 (445) b
	168	5.55 (0.05) b	10.7 (0.65) b	1.14 (0.03) a	9.41 (0.56) a	95.4 (2.25) ab	33.8 (3.97) a	145 (15.1) a	10638 (266) a
	240	5.62 (0.05) b	11.2 (0.70) ab	1.10 (0.07) a	10.2 (0.35) a	102 (2.80) a	28.8 (4.21) a	132 (4.75) a	11242 (171) a
	270	5.59 (0.06) b	12.9 (0.97) a	1.19 (0.04) a	10.9 (1.14) a	105 (4.50) a	32.8 (4.40) a	136 (3.53) a	10896 (227) a
	312	5.56 (0.04) b	10.8 (0.37) b	1.10 (0.06) a	9.91 (0.78) a	105 (8.20) a	29.3 (2.55) a	142 (25.9) a	10478 (155) a
**Sandy soil**	0	6.11 (0.05) a	7.16 (0.84) c	1.13 (0.03) a	6.32 (0.83) c	58.1 (9.62) a	17.5 (3.58) a	129 (16.6) a	3110 (542) b
	168	5.55 (0.13) b	7.83 (0.33) bc	1.09 (0.01) a	7.20 (0.38) bc	65.3 (14.7) a	15.5 (4.46) a	115 (14.7) a	7252 (1123) a
	240	5.32 (0.09) bc	8.99 (0.64) abc	1.08 (0.04) a	8.30 (0.51) ab	57.2 (12.6) a	16.7 (5.52) a	111 (23.5) a	7256 (1067) a
	270	5.24 (0.08) c	9.88 (0.47) a	1.13 (0.03) a	8.71 (0.26) a	63.0 (11.5) a	17.3 (4.44) a	144 (10.8) a	7184 (1112) a
	312	5.19 (0.06) c	9.08 (0.94) ab	1.03 (0.10) a	8.81 (0.40) a	60.9 (9.47) a	14.8 (3.70) a	109 (27.3) a	7371 (1000) a

†Values in the parentheses indicate standard errors.

Different small-case letters within each soil indicate significant differences among N application rates at the 0.05 probability level.

### Soil microbial lipid groups

**Fig 1:**
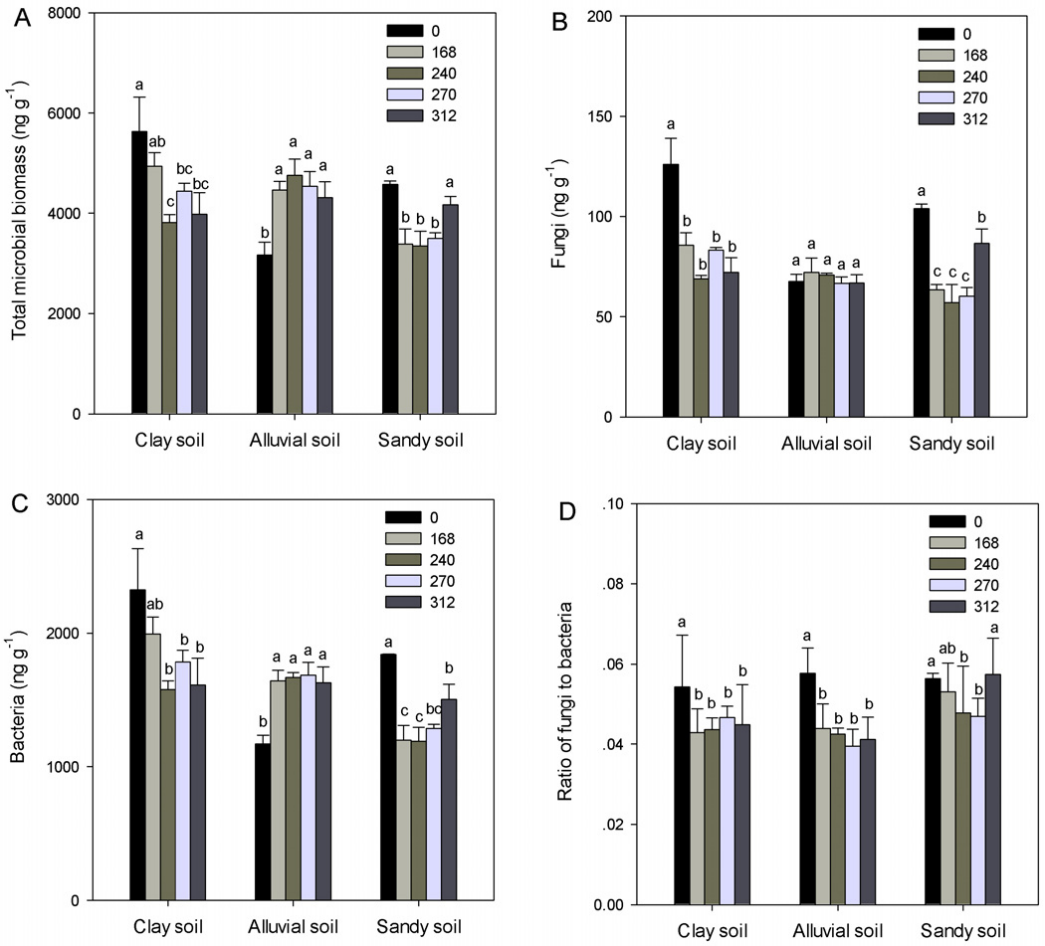
**Concentrations of total microbial biomass (A), fungi (B) and bacteria (C), and ratios of fungi to bacteria (D) in three soils under application of N fertilizers at five rates (0, 168, 240, 270 and 312 kg N ha**^**-1**^**).** Error bars indicate standard errors. Different letters within each soil indicate significant differences among N fertilization treatments.

**Fig 2:**
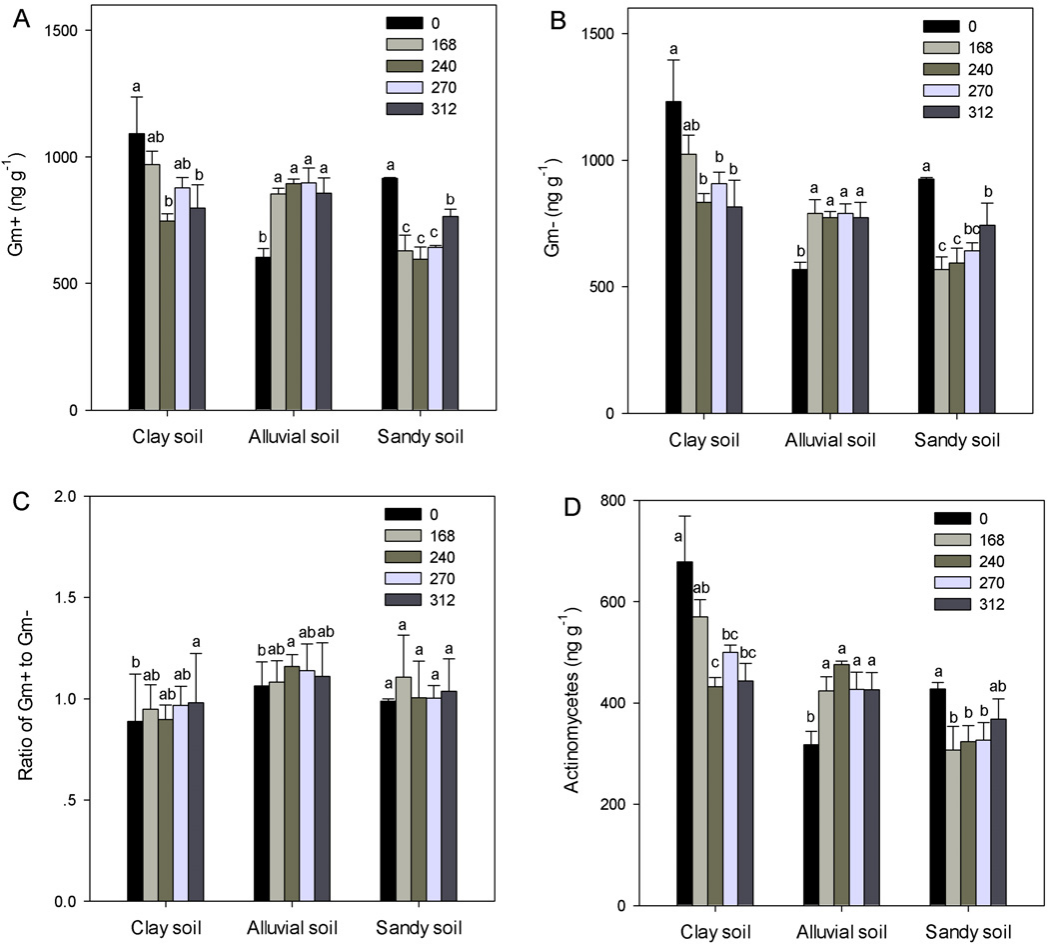
**Concentrations of gram-positive (Gm+, A) and gram-negative bacteria (Gm–, B), ratios of Gm+ to Gm- (C), and concentrations of actinomycetes (D) in three soils under application of N fertilizers at five rates (0, 168, 240, 270 and 312 kg N ha**^**-1**^**).** Error bars indicate standard errors. Different letters within each soil indicate significant differences among N fertilization treatments.

Application of N fertilizers at rates of 240, 270 and 312 kg N ha^-1^ led to significantly lower total microbial biomass in the clay soils compared with the no fertilization treatment (*P* < 0.05, Fig 1). Nitrogen fertilization significantly increased total microbial biomass in the alluvial soils regardless of N application rates (*P* < 0.05, Fig 1). Application of N fertilizers at rates of 168, 240 and 270 kg N ha^-1^ led to significantly lower total microbial biomass in the sandy soils compared with the no fertilization treatment (*P* < 0.05, Fig 1). Total microbial biomass was higher in the clay soils than that in the alluvial soils, which was also higher than that in the sandy soils (*P* < 0.05). Nitrogen fertilization significantly (*P* < 0.05) decreased fungal biomass in both clay and sandy soils but had no effect (*P* > 0.05) on that in the alluvial soils (Fig 1). Application of N fertilizers at rates of 168, 240 and 270 kg N ha^-1^ led to significantly lower fungal biomass in the sandy soils compared with application of N fertilizers at rate of 312 kg N ha^-1^ (*P* < 0.05, Fig 1).The clay soils contained significantly higher fungal biomass than the alluvial and sandy soils (*P* < 0.05). Bacterial biomass responded to N fertilization and soil type similarly as total microbial biomass did (Fig 1). Nitrogen fertilization significantly decreased the ratio of fungal to bacterial biomass in both clay and alluvial soils at all rates and that in the sandy soils at rates of 240 and 270 kg N ha^-1^ (*P* < 0.05, Fig 1). The sandy soils had higher ratio of fungi to bacteria than both the clay and alluvial soils (*P* < 0.05). Gm+, Gm- and actinomycetes generally followed the same trend as bacteria (Fig 2). Compared with the no fertilization treatment, application of N fertilizers at rate of 312 kg N ha^-1^ significantly increased ratio of Gm+ to Gm- for the clay soils, application of N fertilizers at rate of 168 kg N ha^-1^ significantly increased that for the alluvial soils (*P* < 0.05, Fig 2). Ratio of Gm+ to Gm- was highest in the alluvial soils, intermediate in sandy soils, and lowest in the clay soils (*P* < 0.05, Fig 2).

**Fig 3:**
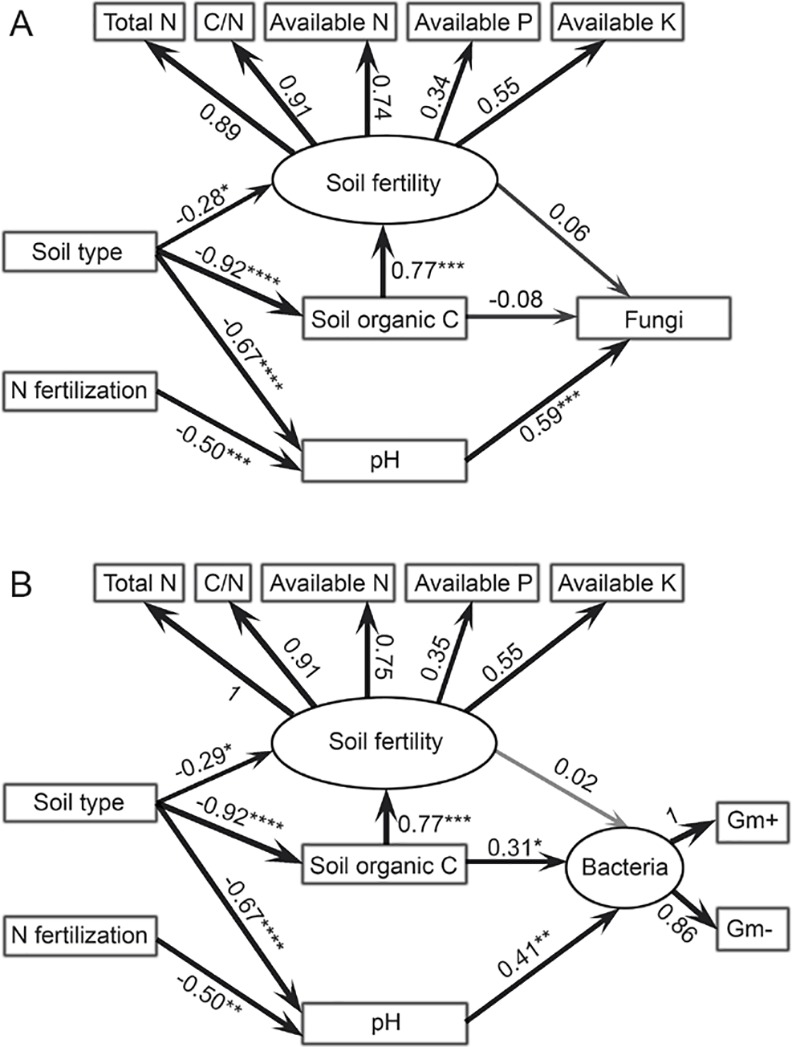
**The structural equation model showing the direct and indirect effects of soil type and N fertilization on fungi (A) and bacteria (B).** Soil fertility index is a patent variable indicated by total N, C/N ratio, available N, P, and K. Bacteria is indicated by Gram-positive (Gm+) and Gram-negative (Gm–) bacteria. The width of arrow indicates the strength of the standardized path coefficient (*, *P* < 0.05; **, *P* < 0.01, ***, *P* < 0.001).

Structural equation modelling was used to assess the direct and indirect effects of soil type and N fertilization on fungal and bacterial biomass (Fig 3). The fitted models for fungal (*χ*^*2*^/df = 1.81, *P* = 0.115, *RMSEA* = 0.063, *GFI* = 0.96) and bacterial (*χ*^*2*^/df = 1.83, *P* = 0.112, *RMSEA* = 0.062, *GFI* = 0.95) biomass met the significance criteria. Both soil type and N fertilization showed significant indirect effect on fungal and bacterial biomass through their influences on soil pH (*P* < 0.05, Fig 3). Soil type also indirectly impacted bacterial biomass through its influence on soil organic C (*P* < 0.05, Fig 3).

### Soil microbial community composition

**Fig 4:**
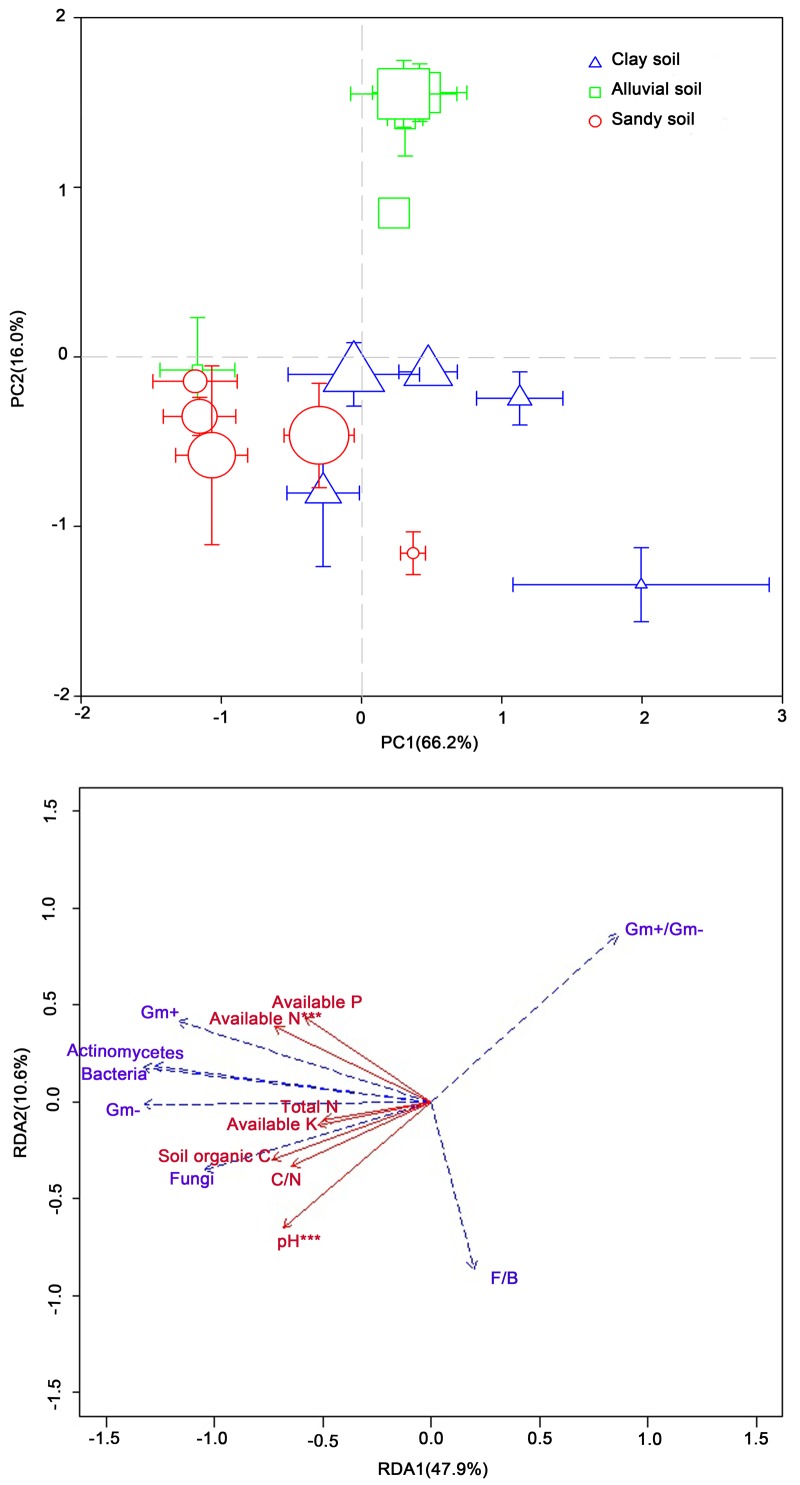
Soil microbial community composition and environmental constraints. A) Principal component analysis (PCA) of the phospholipid fatty acid (PLFA) data. Error bars indicate standard errors. The symbol size is positively correlated with N application rate. B) Redundancy analysis (RDA) of the PLFA data as explained by environmental variables. The explanatory variables followed by asterisks indicate significant influences on the PLFA data (*, *P* < 0.05; **, *P* < 0.01; ***, *P* < 0.001).

Principal component analysis of the PLFA data showed the first principal component (PC1) explained 66.2% and the second (PC2) 16.0% of the total variance in the PLFA data (Fig 4) The non-parametric MANOVA revealed that soil microbial community composition was influenced significantly by soil type (*F* = 51.1, *R*^*2*^ = 0.36, *P* < 0.001), N fertilization (*F* = 7.45, *R*^*2*^ = 0.10, *P* < 0.001) as well as their interaction (*F* = 15.4, *R*^*2*^ = 0.43, *P* < 0.001). Microbial community composition for alluvial soils was significantly different from that for clay and sandy soils (*P* < 0.05, Fig 4). Nitrogen fertilization significantly shifted microbial community composition in all three soils (*P* < 0.05, Fig 4). Differences in microbial community composition between the clay and sandy soils were gradually dismissed with increase in N application rate.

Redundancy analysis of the PLFA data showed that the first and second canonical axes explained 47.9 and 10.6%, respectively, of the total variance (Fig 4). Soil organic C (*P* < 0.001), soil pH (*P* < 0.001), available N (*P* < 0.001) and available K (*P* < 0.01) all played a significant role in shaping soil microbial community composition (Fig 4).

## Discussion

### N fertilization effect

Application of N fertilizers significantly decreased soil pH regardless of soil type. This is well documented and mainly resulted from soil processes which produce protons, including oxidation of ammonium to nitrite and then to nitrate as well as nitrification [14]. A survey across China suggested that 8 to 25 years of N fertilization led to decreases in soil pH by 0.45–2.20 units [28]. The pH decreases by N fertilization was moderate in both clay and alluvial soils of our study, probably due to the short duration of these two experimental fields. The greatest N-induced pH decreases was observed in the sandy soils, which suggests these soils had a lower buffering ability than the clay and alluvial soils. We also observed that soil pH decreased significantly with increase in N application rate in the sandy soils, which is consistent with several other studies that have shown a clear relationship between acidification and N application rate [29]. The reason for this is probably related to the fact that the neutralizing effect of nitrate uptake by plants was reduced when excess N was applied [30].

Nitrogen fertilization significantly decreased microbial biomass in the clay and sandy soils. This is in line with several other studies which found repeated application of N fertilizers reduced soil microbial biomass compared to the control [10]. These results are probably related to soil pH as supported by both structural equation modeling and RDA results which suggested that N fertilization influenced soil microbial communities mainly through its effect on soil pH. Although soil microorganisms could use urea as nitrogen source, they could also be inhibited due to toxicity of ammonia when urea was applied at high rates [11]. However, the reduction in fungal and bacterial biomass by N fertilization was not synchronous, which resulted in a significant decrease in the ratio of fungi to bacteria in the N-fertilized soils compared with the non-fertilized soils. It has been reported that fungi are more sensitive to pH changes than bacteria [31]. Therefore, fungal biomass decreased faster than bacterial biomass with pH decreases induced by N fertilization, especially given that the pH ranges in our study are more beneficial to bacteria than fungi [32].

It is expected that N fertilization significantly increased maize yields in three studied soils. However, the fertilization-induced increase in maize yields was not correlated with N application rate in our study. There were no significant differences in maize yields among N treatments with different application rates. This is exciting because farmers usually apply N fertilizers at rate of 270 kg N ha^-1^ to achieve high yields, which seems in our study could be achieved by applying N fertilizers at rate of 168 kg N ha^-1^. In addition, soil microbial biomass and community composition were generally similar in soils with N applied at rate of 168 kg N ha^-1^ compared with that in soils with N applied at rates of 240, 270 and 312 kg N ha^-1^. This means that farmers should apply N fertilizers at rate of 168 kg N ha^-1^, which could achieve high maize yield on one hand while maintain soil microbial functions on the other hand.

### Effect of soil type

Soil type has been recognized as an important determinant of soil microbial biomass and community composition [33]. In this study, the clay soils contained significantly higher total microbial biomass than the alluvial soils, which also had significantly higher that than the sandy soils. This is probably associated with soil organic C as soil microorganisms are usually considered to be C limited [34]. This is supported by the structural equation modeling results which showed soil type indirectly influenced bacteria through its effect on soil organic C. Soil texture may also contribute to the observed results since it controls aeration and water conditions which exert significant effect on soil microbial communities [35]. It is interesting that the response of fungal and bacterial biomass to N fertilization in the alluvial soils was quite different from that in both clay and sandy soils. Nitrogen fertilization did not influence fungal biomass but significantly increased bacterial biomass in the alluvial soils. This observation was probably related to the significant differences in microbial community composition of the alluvial soils as supported by the PCA results. It could be that the alluvial soils have some special microorganisms that can tolerate high ammonia concentrations induced by N fertilization [36].

## Conclusion

In this study, we examined the effects of different N application rates on microbial communities in three types of soils. We found that application of N fertilizers in the clay and alluvial soils significantly decreased microbial biomass regardless of N application rate, which was probably related to soil pH decreases in these soils. We also observed that N fertilization significantly increased bacterial biomass in the alluvial soils, which was attributed to microbial toleration to high ammonia concentrations. However, there were generally no significant differences in soil microbial communities among treatments with N application. Given that maize yields were also similar among treatments with N application, we concluded that the current N fertilization strategy in the study area should be switched to a more environmentally friendly one, which could maintain current crop yields while reduce application of mineral fertilizers. This is important because soil sustainability could be largely retained when less N fertilizers were applied.

## Supporting Information

S1 File Original data set used in this manuscript. (PDF)
