# Supplementary material for: Decreasing Nitrogen Fertilizer Input Had Little Effect on Microbial Communities in Three Types of Soils
Source: PLoS One. 2016 Mar 18;11(3):e0151622. doi: 10.1371/journal.pone.0151622 (PMC4798769; doi:10.1371/journal.pone.0151622)
Supplement: S1 File — (PDF) [file pone.0151622.s001.pdf]

Soil type	N Treatment	pH	SOC	TN	C/N	Available N
Clay soil	0	6.10	16.41	1.39	11.81	135.10
	0	6.16	19.60	1.44	13.59	146.30
	0	6.14	16.50	1.45	11.35	100.80
	168	5.91	16.47	1.46	11.26	140.70
	168	5.99	16.98	1.38	12.29	126.00
	168	6.05	16.91	1.50	11.24	121.10
	240	6.04	17.10	1.41	12.09	127.40
	240	5.95	16.47	1.47	11.19	130.20
	240	5.89	16.60	1.40	11.84	126.70
	270	5.83	17.05	1.37	12.41	131.60
	270	5.92	16.05	1.44	11.19	135.10
	270	5.96	15.84	1.38	11.46	124.60
	312	5.86	13.63	1.46	9.32	137.20
	312	5.81	13.21	1.45	9.08	135.80
	312	6.00	14.10	1.38	10.23	134.40
Alluvial soil	0	5.88	12.08	1.20	10.08	83.30
	0	5.79	12.73	1.19	10.65	88.20
	0	5.72	13.86	1.15	12.04	88.20
	168	5.59	11.25	1.16	9.71	98.00
	168	5.49	10.82	1.11	9.77	93.80
	168	5.56	9.97	1.14	8.76	94.50
	240	5.68	10.83	1.11	9.77	99.40
	240	5.58	12.02	1.17	10.30	102.20
	240	5.61	10.78	1.03	10.44	105.00
	270	5.66	11.97	1.23	9.75	102.90
	270	5.56	12.92	1.19	10.89	101.50
	270	5.54	13.92	1.16	12.03	109.90
	312	5.53	10.54	1.16	9.05	101.50
	312	5.54	11.24	1.06	10.58	98.70
	312	5.61	10.70	1.06	10.10	114.10
Sandy soil	0	6.09	7.37	1.16	6.35	68.60
	0	6.07	7.88	1.10	7.13	49.70
	0	6.16	6.23	1.14	5.47	56.00
	168	5.68	8.20	1.08	7.61	60.20
	168	5.54	7.73	1.08	7.14	53.90
	168	5.42	7.57	1.10	6.86	81.90
	240	5.31	9.73	1.10	8.83	56.70
	240	5.24	8.66	1.11	7.81	70.00
	240	5.42	8.57	1.04	8.26	44.80
	270	5.15	9.88	1.15	8.58	76.30
	270	5.28	9.40	1.10	8.54	56.70
	270	5.30	10.35	1.15	9.01	56.00
	312	5.17	8.03	0.91	8.80	70.70
	312	5.25	9.35	1.10	8.46	51.80

	312	5.14	9.85	1.08	9.16	60.20

Soil type	N Treatment	Available P	Available K	Total biomass	Fungi	Gm+
Clay soil	0	42.22	217.42	6225.91	137.83	1206.19
	0	34.01	186.27	5792.26	128.26	1142.00
	0	21.05	182.25	4877.21	111.90	930.44
	168	34.55	193.30	4836.76	83.00	956.31
	168	51.81	172.21	5246.95	92.74	1029.24
	168	27.48	188.28	4745.67	80.91	924.59
	240	33.95	187.28	3650.90	69.02	715.06
	240	46.10	196.32	3973.96	70.52	768.33
	240	28.65	225.45	3813.96	66.96	756.51
	270	29.25	156.13	4408.42	81.67	873.96
	270	73.66	178.23	4616.26	83.81	921.61
	270	33.93	190.29	4324.97	83.93	839.54
	312	27.75	178.23	4481.59	78.93	905.29
	312	40.70	203.35	3717.70	64.26	737.15
	312	28.51	173.21	3743.75	73.11	751.14
Alluvial soil	0	11.25	144.08	2881.02	63.63	562.29
	0	30.70	190.29	3274.05	68.41	624.65
	0	31.64	183.26	3348.38	70.59	621.55
	168	37.24	153.12	4666.94	78.51	881.06
	168	34.60	128.00	4367.31	64.72	836.52
	168	29.44	155.13	4380.74	73.32	844.83
	240	30.41	136.04	4690.77	69.76	878.70
	240	31.89	134.03	4477.38	71.46	892.69
	240	23.97	127.00	5114.74	71.21	915.20
	270	31.63	133.03	4205.28	62.72	829.14
	270	37.66	140.06	4725.55	68.19	942.31
	270	29.11	136.04	4697.34	68.65	921.15
	312	32.00	146.09	4125.25	62.15	831.35
	312	26.92	113.94	4682.72	68.47	926.80
	312	29.06	165.17	4152.87	70.01	815.39
Sandy soil	0	20.28	145.08	4566.20	106.22	912.32
	0	13.43	111.93	4656.36	103.82	917.66
	0	18.67	131.02	4517.36	101.88	919.02
	168	11.83	101.88	3731.43	66.43	700.70
	168	20.47	131.02	3252.96	61.87	591.78
	168	14.22	112.93	3159.71	61.72	593.30
	240	17.35	138.05	3076.49	50.61	546.17
	240	21.82	98.87	3658.46	67.30	641.18
	240	10.85	95.86	3300.49	53.23	601.74
	270	18.49	139.05	3595.67	59.96	643.31
	270	21.08	156.13	3376.44	55.92	635.24
	270	12.42	136.04	3532.27	64.70	650.57

312	18.92	93.85	4328.66	93.66	796.86
312	11.83	140.06	4185.59	86.63	751.00
312	13.59	91.84	3985.10	79.35	744.49

Soil type	N Treatment	Gm-	Bacteria	Actinomycetes	F/B	Gm+/Gm-
Clay soil	0	1366.98	2573.17	757.40	0.05	0.88
	0	1279.23	2421.23	698.27	0.05	0.89
	0	1049.60	1980.05	579.20	0.06	0.89
	168	990.72	1947.03	548.80	0.04	0.97
	168	1109.34	2138.58	609.03	0.04	0.93
	168	971.60	1896.19	551.58	0.04	0.95
	240	795.74	1510.80	433.35	0.05	0.90
	240	869.42	1637.75	450.00	0.04	0.88
	240	831.51	1588.02	414.79	0.04	0.91
	270	893.71	1767.67	484.29	0.05	0.98
	270	959.13	1880.73	512.63	0.04	0.96
	270	870.68	1710.22	503.92	0.05	0.96
	312	933.92	1839.21	483.80	0.04	0.97
	312	726.51	1463.67	425.26	0.04	1.01
	312	784.93	1536.07	421.22	0.05	0.96
Alluvial soil	0	532.25	1094.54	289.67	0.06	1.06
	0	580.30	1204.95	342.09	0.06	1.08
	0	587.75	1209.30	320.69	0.06	1.06
	168	848.78	1729.84	392.51	0.05	1.04
	168	742.33	1578.85	434.00	0.04	1.13
	168	778.36	1623.19	445.57	0.05	1.09
	240	774.72	1653.43	481.12	0.04	1.13
	240	748.17	1640.86	468.64	0.04	1.19
	240	795.42	1710.63	478.66	0.04	1.15
	270	748.91	1578.05	387.52	0.04	1.11
	270	826.20	1768.51	447.28	0.04	1.14
	270	789.14	1710.28	446.33	0.04	1.17
	312	724.97	1556.31	411.15	0.04	1.15
	312	839.85	1766.65	465.31	0.04	1.10
	312	751.63	1567.02	402.96	0.04	1.08
Sandy soil	0	932.87	1845.19	437.39	0.06	0.98
	0	923.62	1841.28	413.32	0.06	0.99
	0	921.71	1840.73	432.40	0.06	1.00
	168	623.68	1324.37	360.63	0.05	1.12
	168	553.74	1145.52	284.14	0.05	1.07
	168	526.42	1119.72	276.46	0.06	1.13
	240	544.40	1090.57	293.96	0.05	1.00
	240	658.64	1299.82	356.21	0.05	0.97
	240	578.49	1180.22	320.16	0.05	1.04
	270	677.77	1321.08	363.86	0.05	0.95

270	627.48	1262.72	294.18	0.04	1.01
270	618.56	1269.13	319.67	0.05	1.05
312	819.48	1616.33	409.66	0.06	0.97
312	760.70	1511.70	365.30	0.06	0.99
312	647.52	1392.01	328.83	0.06	1.15
